# Using large-scale community science data and computer vision to evaluate thermoregulation as an adaptive driver of physiological color change in *Anolis carolinensis*

**DOI:** 10.1186/s12983-025-00580-4

**Published:** 2025-10-09

**Authors:** Serena Price, Robert Guralnick, Coleman M. Sheehy, Jacob Idec

**Affiliations:** 1 Division of Biodiversity Informatics, Florida Museum of Natural History, University of Florida, Gainesville, FL 32611 USA; 2 Division of Herpetology, Florida Museum of Natural History, University of Florida, Gainesville, FL 32611 USA

**Keywords:** Camouflage, Community science, Computer vision, Physiological color change, Social signaling, Thermoregulation

## Abstract

**Background:**

Facultative, physiological color change has many potential adaptive functions, and the ability of the green anole (*Anolis carolinensis*) to shift between brown and green coloration is no exception. Three non-mutually exclusive hypotheses for such color changes include: 1) The camouflage hypothesis, which states that individual anoles use brown and green coloration to blend into their background; 2) The social signaling hypothesis, which states that coloration shifts convey intraspecific signals such as dominance, submission, and mating status during interactions; 3) The thermoregulation hypothesis, which states that shifting to darker brown coloration during colder temperatures allows for increased absorption of solar radiation as heat.

**Results:**

We showcase the utility of a computer vision pipeline to derive individual-level color (green versus brown) from a large dataset of citizen science observations spanning the southeastern USA. We used this color information along with climate, seasonal timing information and background in images to test associations between color morph, temperature and time of year. Results show that brown-presenting *A. carolinensis* were observed more frequently at lower temperatures during winter. However, the observed correlation between presenting color and temperature was absent during the summer breeding season. We did not find strong evidence for background color matching.

**Conclusion:**

We found support for both the thermoregulatory hypothesis and social signaling hypothesis dependent on time of year, which suggests multiple independent drivers are influencing physiological color changes in *A. carolinensis.* Further, this work shows the power of leveraging large-scale digital field images and machine learning to derive insights about how species can regulate phenotype to maintain their thermal and biotic niche optima.

## Background

The animal kingdom is rich with species capable of changing colors rapidly from one state to another, including many diverse ectothermic vertebrate taxa such as fishes, amphibians, and reptiles [1]. The mechanisms for these color changes are thought to be relatively conserved among vertebrate taxa and to involve pigments rapidly translocating in chromatophores via neuroendocrine signaling [2]. The adaptive value of these physiologically-mediated color changes has long been debated and the most common, non-mutually exclusive hypotheses are facultative crypsis, intraspecies social behavior, and thermoregulation [3]. Each of the three hypotheses has received support from studies involving reptiles. For example, many species of chameleons in the genus *Chamaeleo* utilize color change primarily for interspecies communication, while other, relatively closely related species likely change color in order to better thermoregulate [4, 5]. In other groups, such as *Anolis carolinensis*, the purpose of physiological color change continues to be hotly debated due to conflicting evidence from different studies.

*Anolis carolinensis*, commonly known as the green anole, is a diurnal lizard species in the family Anolidae capable of facultative, physiological color change. Dorsal coloration can shift rapidly between distinct shades of green and brown, and shifts are accomplished through melanophores, a kind of chromatophore that translocates melanin pigment [6]. Early studies suggest that the change in coloration serves as crypsis in varying levels of light [7]. Later studies challenged this, instead hypothesizing that facultative color change is a form of social behavior to signal dominance [8]. Lastly, there is debate on whether facultative color change may have a thermoregulatory purpose. In general, darker ectotherms have been shown to more effectively utilize environmental sources of heat through the absorption of solar radiation and conductive heat transfer than lighter ones, leading to higher body temperatures [9]. Earlier studies note a link between temperature and color in many species in the genus *Anolis*, [6] and it has been hypothesized that the lower the air temperature, the more anoles should be brownish, but more recent work finds little or no evidence of this in *A. carolinensis*. [10] In sum, it is unclear whether color change has a thermoregulatory function, and the overall strength of support for any of these hypotheses is also unresolved. We also note that none of these hypotheses are mutually exclusive.

Much of our knowledge about drivers of physiological color change has come from studies performed on a small sample of male *A. carolinensis* in a laboratory setting. While these laboratory approaches are useful for controlling for exogeneous variables, they also may not represent real-world environments and how populations respond in the wild [11]. Broadening the scope of the animals we observe across space and time could shed further light on the function of physiological color change in wild populations. However, field-based studies have typically been limited in spatial and temporal extents and, depending on the design of the study, may offer limited potential for generalizability [12]. Recently, however, the combination of rapidly growing citizen science field photographs, such as from iNaturalist (www.inaturalist.org/), as well as new advances in computer vision and AI (Artificial Intelligence) have provided a means to scale up extracting key traits and studying processes in natural systems. Key examples include documenting color polymorphisms in salamanders [13] and flower color for North American wildflowers [14]. These advances may provide a means to test primary drivers of physiological color change in *A. carolinensis*.

Here we addressed key hypotheses regarding the drivers of physiological color change in the green anole, *Anolis carolinensis*. We did so by first utilizing computer vision approaches applied to community science observations to evaluate proportions of color morphs across a wide region, covering much of the native range of green anoles. We then used daily climate data to determine temperature at the time each field photograph was captured. This information, along with known timing of the breeding season provided the basis for directly testing two primary hypotheses: 1) brown coloration should occur more frequently in cooler temperatures in late Fall through Spring in order to increase absorption of solar radiation and 2) during breeding season, green and brown colorations should primarily facilitate social dominance structures associated with attracting mates and defending territories. Finally, we post-hoc examined if there is evidence for color change potentially serving a role in crypsis, focusing on whether there is evidence for matching between brown and green coloration and background.

## Methods

### Data acquisition

We downloaded Research-Grade observations of *Anolis carolinensis* from iNaturalist (accessed 12 December 2023) for 20 counties in 7 states located around major cities, spanning much of the range of the species. We chose to analyze data from counties containing major metropolitan areas to maximize sample sizes for spatiotemporal analysis. Restricting the dataset to urban areas helps avoid bias introduced by differing sampling regimes, as rural zones often have fewer observations and less consistent temporal coverage. Counties with major cities were selected to incorporate a range of climates and geographical locations while still providing reasonable sample sizes for analyses. A total of 28,634 images were downloaded across the 20 counties using the “rinat” R package [15].

### Image segmentation

The downloaded images were segmented using the foundational machine learning approach groundedSAM, which uses the groundingDINO zero-shot object detector to identify bounding boxes for objects in images and SegmentAnything to segment objects from background given a bounding box [16, 17]. Using the groundingDINO text prompt “subject” we obtained segment candidates for 28,275 images. We used liberal text and box threshold parameters of 0.25 to start, which may lead to false positives but limits false negatives. Despite the generality of groundedSAM, given the dramatic variation in angle and background in iNaturalist images, many of these segment candidates did not properly capture all of the anole visible in the image. To highgrade acceptable anole segments in a single manual step, we clustered the segments by their features extracted using a pre-trained neural network. This approach groups segments that are similar in high-dimensional image space together, and since fully visible anoles are similar in shape, these naturally group together. Our clustering approach used AffinityPropagation with 10 clusters. We examined samples from these groupings and kept those groups with samples containing only acceptable anole segments, discarding the rest [18, 19]. A final examination of a test set of images from Alachua County, FL was conducted to quantify clustering effectiveness and to remove any remaining incorrect image segments. See Results for the final numbers of segments.

### Color categorization

**Fig. 1:**
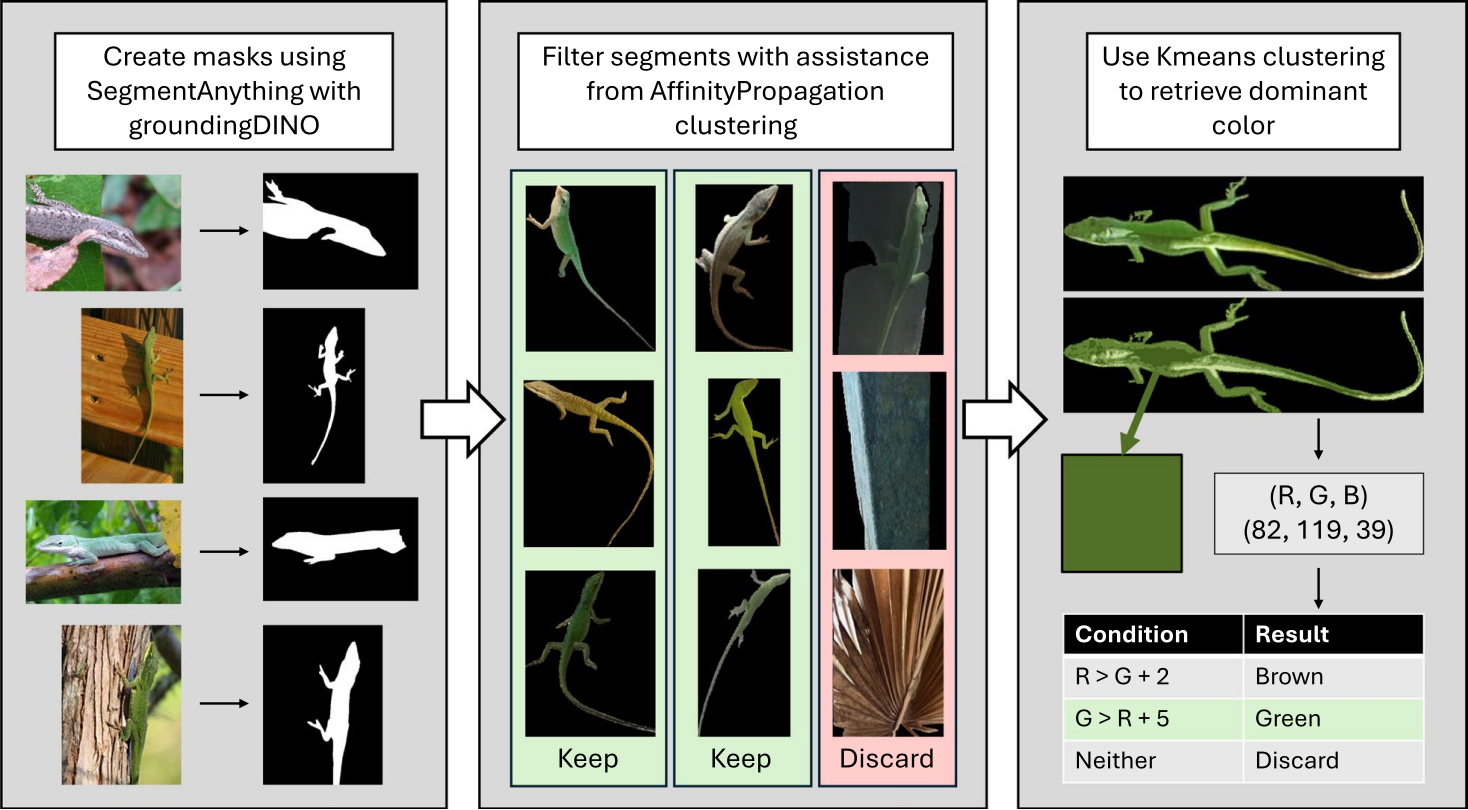
Visualization of the computer vision pipeline used to determine the dominant color in iNaturalist observation images for green anoles (*Anolis carolinensis*) used in this study. First, the anole is segmented from the image using SegmentAnything with GroundingDINO to create a mask. Then, the masks are filtered to remove inaccurate segments using the AffinityPropagation clustering algorithm. To retrieve the dominant color of the anole, Kmeans clustering is used to quantize the image and report the color that involves the largest number of pixels in the segment. The retrieved RGB value is evaluated to determine if the anole is green, brown, or inconclusive

Given the scale of the task of labeling images green or brown, we opted for an automated approach. The color of each *A. carolinensis* observation was determined by using the K-means clustering algorithm in the “scikit” Python package to find the most dominant color of the image, out of four clusters, as a RGB (Red Green Blue) value [20]. The most dominant color is defined as the RGB value of the cluster that takes up the largest proportion of the image. (Fig. 1). Then, each image was labeled as either green or brown using an RGB threshold. We defined green-presenting anoles are those where the dominant color’s G > R + 2, and brown-presenting anoles are defined as observations where R > G + 5. The clustering and thresholding values were iteratively determined using a test set of 507 labeled images, with an accuracy of 93% when compared to a human validated dataset. Observations where neither condition was met, representing 19% of the total segmented anole images, were considered “unclear” and removed from the dataset. (Fig. 1). We opted for using K-means clustering rather than averaging RGB values because the latter resulted in a higher number of images categorized as “unclear.” The final number of observations in the dataset after segmentation, clustering, and labeling was 13,006 observations.

### Temperature data assembly

To investigate associations between temperature and color morph, we first derived temperature data for each observation based on the day, hour, and geographic location at which it occurred. To reduce computational demand, and get a reasonable approximation of the climate context, the location of each observation was assigned as the 25 km grid cell into which the observation fell using a regularly spaced grid over the range of the data. We then obtained the average daily minimum and maximum temperatures for each cell location and day of the year in which it occurred from the PRISM (Parameter-elevation Regressions on Independent Slopes Model) dataset using the “prism” R package [21]. This provided each observation with a minimum and maximum temperature value based on the actual day it was recorded and the 25 km grid cell in which it was located.

We then estimated hourly temperatures using the stack_hourly_temps() function from the chillR package [22]. This function models diel temperature variation using a sine curve for daytime warming and a logarithmic decay function for nighttime cooling. Given the min and max temperature, location, day of the year, and hour of an observation, we used this function to calculate the hourly temperature corresponding to the time of the observation. This allowed us to approximate the ambient temperature at the place and time that the observation was made.

### Background matching data assembly

To test for cryptic color selection in *A. carolinensis*, we compared the dominant RGB color value of the segment to the dominant RGB color value of the background immediately around the segment using a 607 image set drawn from the full set of clustered images. Background color was determined by making a 20 pixel contour around each mask, then repeating the steps for dominant color retrieval used for the segments. To compare the two dominant colors, we calculated a greenness percentage index for both the anole and the background using the formula G/(R + G + B). This allows for comparison between the background and the anole without having to categorize the background into specific colors, which may not always be green or brown.

### Modeling of seasonal effect on color

To assess the relationship between season and color selection, we fitted a cyclical GAM (General Additive Model) week of the year and latitude as predictors using the R package “mgcv.” [23] The response variable was the binary color morph classification (green vs brown), modeled as a binomial outcome. To estimate the interactive effect of week of year and latitude, we used a tensor product smooth modeling week as a cyclic cubic spline and latitude as a thin plate spline with the smoothing dimension k set to 3. We then compared this interaction model to two simpler alternatives: one model including only week of year without the latitude effect, and another including additive effects of week of year and latitude without interaction. Models were compared using Akaike Information Criterion (AIC) scores. To visualize the interaction model, we predicted values over the full year (0–52 weeks) at the 10th, 50th, and 90th percentiles of latitude in the dataset. Predictions were made on the link (logit) scale and back-transformed to probabilities, allowing us to display the estimated proportion of green individuals across time and space with 95% confidence intervals.

### Modeling of temperature effect on color

To evaluate the relationship between temperature and color expression, we fit a binomial logistic regression using base R’s glm() function. The model, visualized using the R package “ggplot2,” [24] predicted binary color morph (green vs. brown) from approximate hourly temperature with an interaction term representing breeding vs. non-breeding season. Observations from 1 April through 30 September, an approximation of the summer breeding season in *A. carolinensis*, were classified as belonging to the breeding season, while all other months belonged to the non-breeding season [25]. We used this approximation because it is known that breeding season varies minimally in peak timing and duration across latitude, with timing from about April to September in the Carolinas and Virginia [26], and from about March to September in Southern Florida [27]. As with the seasonal model, we compared this interaction model to two simpler alternatives: one model including only temperature without the breeding vs. non-breeding season effect, and another including an additive effect of breeding vs. non-breeding season without interaction. Models were compared using Akaike Information Criterion (AIC) scores. For visualization, predictions were made on the logit scale and back-transformed to probability using the inverse logit function. Confidence intervals were calculated using standard errors from the model and visualized as shaded ribbons around the fitted curves. This allowed us to directly compare how temperature influences green coloration between the breeding and non-breeding season.

### Modeling of potential background-matching

We evaluated the potential for background-matching using a univariate linear regression where anole segment greenness percentage (see above for calculation of this index) was a response variable and immediate background greenness percentage index, calculated the same way as for the anole index, was used as a predictor. All models were fit using the lm() function from R’s base statistical tools. A strong, positive significant relationship would indicate that there is an association between coloration of anole and background coloration. To verify that this association is not a confounder for our temperature results, we also assessed whether background greenness was significantly correlated with temperature.

## Results

### Data retention & validation

**Fig. 2:**
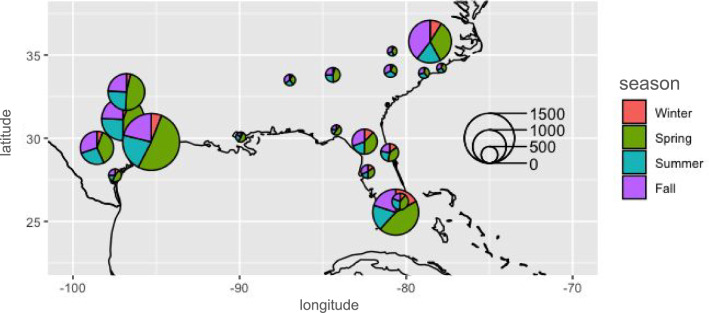
Spatial and seasonal distributions of iNaturalist observation data for green anoles (*Anolis carolinensis*) used in this study. Each pie is one of the 20 populous counties within the species’ range selected for the study. The states represented by these counties are Alabama, Georgia, Florida, Louisiana, North Carolina, South Carolina, and Texas. The size of the pie shows the number of observations coming from that county, and the size of the pie slices show the proportion of observations for each county coming from each season. Spring observations were the most frequent in every county, while winter observations were the least frequent

Out of the 28,634 *Anolis carolinensis* observations retrieved from iNaturalist, 11,677 records were retained after segmentation, clustering, labeling, and temperature approximations to form the final data set. (Fig. 1). The majority of the retained observations were recorded in Texas, Florida, and North Carolina, with spring being the season with the most observations (Fig. 2). These three states cover the northern, southern, and western extremes of the range of this species [28]. Human verification of segments post-clustering from the test set found that the dataset still retained a small percentage (2%) that likely represented partial photos or poor angles. We deemed this error rate acceptable for further downstream analysis. We also validated color clustering, and the differentiation between brown and green anoles was accurately determined at a rate of > 93% in a sample set of 507 segmented images from Alachua County, Florida. We considered this success rate to be sufficiently high for downstream modeling.

### Results of seasonal models

**Fig. 3:**
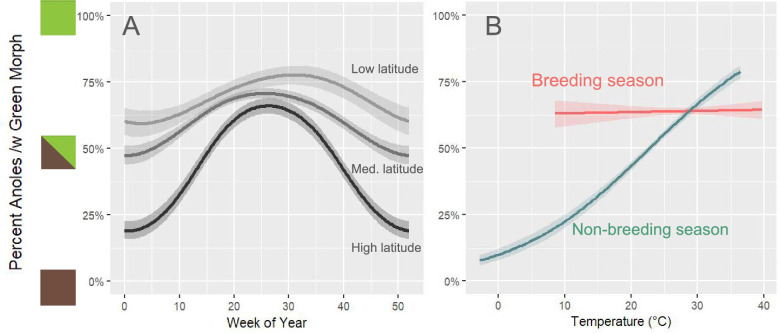
Seasonal and thermal predictors of the percent of green anoles (*Anolis carolinensis*) observed presenting the green color morph. Panel A) Predicted probability of an anole being green across weeks of the year, modeled using a generalized additive model with a cyclic spline for week and controlling for latitude. Low latitude is the 10th quantile of latitude in the data, medium is the 50th quantile, and high is the 90th quantile. Panel B) Predicted probability of an anole being green across observed temperatures, modeled using a binomial generalized linear model. Separate models are presented for summer (breeding season) and non-summer (non-breeding season). Shaded areas in both panels show 95% confidence intervals

We fit cyclical GAMs with brown or green color morph as the response variable and week of year and latitude of the observation as the predictors. Of the three models we evaluated in model selection, the best model (based on comparisons of AIC scores) included an interaction between week of year and latitude (ΔAIC = − 77 between best model and second best model). In this best model, the smooth term of the interaction between week of year and latitude was a highly significant predictor (*p* < 0.001). The adjusted pseudo-R2 (using the method in mgcv [23]) was 0.077 and the deviance explained was 5.93%, values we consider reasonable given the many other factors likely influencing anole color expression. The best model showed an overall relationship where the green increases during the summer and decreases in the fall and winter (Fig. 3). However, the meaningful latitude effect captures a relationship where the curve representing the percentages of green over the year is higher (more green) and flatter (less variation in green over the year) at lower latitudes (Fig. 3). Conversely, at higher latitudes the curve is lower (less green, more brown) and also steepens, indicating much greater seasonal variation in high latitudes with cold winters.

### Results of temperature models

We fit logistic regressions with brown or green color morph as the response variable and estimated hourly temperature and breeding vs. non-breeding season of the observation as the predictors. Of the three models evaluated, the best model (based on comparisons of AIC scores) included an interaction between temperature and breeding vs. non-breeding season (ΔAIC = − 150 between the best and second best model). The percent deviance explained by this model was 3.8%. In observations recorded during the non-breeding season (n = 4300), there is a significant relationship between green color presentation and temperature (*p* < 2e-16)—green is present more often when temperatures are warmer and brown is present more often when temperatures are cooler. Conversely, observations recorded during the breeding season (n = 7350) show no significant relationship between the two variables (*p* = 0.29), instead indicating that anoles consistently show green in the summer regardless of temperature.

### Results of potential background matching models

We evaluated potential background-matching using a univariate linear regression predicting anole greenness with background greenness via greenness indices. We found a significant relationship (*p* < 2e-16) but marginal effect size (R-squared = 0.120). We found background greenness to be weakly correlated with temperature (correlation coefficient = 0.08), and a linear model with background greenness as a predictor of temperature was not significant (p = 0.069), indicating that it is not a confounder for our temperature results.

## Discussion

We investigated the hypothesis that physiological color change in *Anolis carolinensis* is functional for both social signaling and thermoregulation by modeling how temperature in breeding vs. non-breeding seasons is associated with color morph expression. Key to this effort was leveraging the scale of citizen science data to capture broad environmental and latitudinal gradients across much of the range of the species. Our model results support both a thermoregulatory and social signaling role in the shift from brown to green coloration, showing that physiological color change in *A. carolinensis* may serve multiple functions, dependent on time of year. Below we further discuss how examining color change responses across seasons may be essential for fully understanding causes and consequences of facultative color change more broadly.

### Multiple drivers of seasonal color shifts

As predicted, we found a strong relationship between the proportion of green or brown anoles and the week of the year. Green coloration is significantly more common during the summer breeding season, and in the non-breeding season there is a sharp decline in green coloration until brown becomes the dominant color, corroborating the generality of the well-supported social breeding behavior hypothesis [10, 29, 30]. However, the low latitude populations of *A. carolensis* have much weaker trends over the season between predominantly green and brown than those at higher latitudes (Fig. 3). Subsequent models with temperature as the key predictor help explain these differential trends, showing that decreased temperature is strongly associated with a higher percentage of brown color expression, but only in the non-breeding season from October to March.

While our focus here is in *A. carolinensis,* tradeoffs between social signaling, thermoregulation and camouflage likely also occur in other species, as has been demonstrated in the fiddler crab *Uca pugilator*, where the usage of physiological color change may be a tradeoff between thermoregulatory behaviors and mating behaviors that vary between males and females [31]. In chameleons, the social signaling hypothesis is now generally favored over the previous camouflage hypothesis, but both may be relevant depending on timing and context [5]. In *A. carolinensis*, seasonal and life history context are likely both important, with strong physiological color change responsiveness to temperature in the Fall and Winter non-breeding season, but no response during the late Spring and Summer breeding season. More generally, continuing work to uncover the multifaceted uses of physiological color change may lead to re-evaluation of single-driver hypotheses in favor of context-dependent trade-offs.

While it can be difficult to control confounding variables in observational studies, we believe this seasonal result to be robust because the breeding season of *A. carolensis* is relatively stereotyped and varies only minimally across latitude (see Methods above). The consistent seasonal pattern we found across the range of *A. carolensis* demonstrates that non-temperature cues such as photoperiod, seasonal hormonal changes, and social context are associated with a high percentage of green anoles in the summer across the entire region, even in higher latitudes where brown is widely expressed in the winter. The lack of a temperature effect during the breeding season may indicate that social signaling takes priority over thermoregulation, such that even in cooler summer periods anoles remain green. It is also possible that summers in the southeastern USA are generally warm enough that shifting to brown coloration for thermoregulation is simply less important.

Finally, we explored the potential role of physiological color change in crypsis. We found a significant but weak positive association between anole greenness and background greenness; ~ 12% of the variation in anole greenness is explained by background. However, it is difficult to assess whether this weak correlation may itself relate to issues with uncontrolled lighting and different camera sensors. These are factors that cannot easily be controlled in citizen science field images, and which could create artificial autocorrelation. We also directly tested if background correlates strongly with temperature, which would suggest potential for confounding the signal between temperature and anole coloration, and found that background and temperature were not significantly correlated. Taken as a whole, we argue there is only limited evidence of a role for background color matching. However, we do see a need for further work in more controlled settings to test the importance of crypsis in physiological color change in *A. carolinensis*. Such work could help clarify the background matching hypothesis and identify the contexts in which it may be important.

### Value and shortcomings of big data approach and overall conclusions

By comparing the breeding and non-breeding seasons through large-scale data analysis, we are able to derive evidence for a multi-faceted set of color change responses in *A. carolensis* that are contextual across seasons. In particular, our use of a latitudinal gradient in temperature was crucial for insights into the likelihood of color change under different temperature regimes at similar times of year. This macroscale view on facultative color change only partially aligns with previous studies examining drivers of physiological color change in *A. carolinensis*. [6, 8, 10, 30] Based on laboratory studies, thermoregulation has thus far been poorly supported, but the timing of both experimental and observational studies may be confounding, as studies are often conducted during the summer breeding season [10, 30]. While we argue our results show a clear association between ambient air temperature and coloration, associational studies cannot directly test proximal causes, nor can we directly account for behavioral aspects of thermoregulation, such as posturing, that may enhance or reduce the heat gained from solar radiation. Even so, these results suggest new pathways for exploring causation: for example, through experiments that account for season or manipulate seasonal cues such as photoperiod, habitat context, behavior, and temperature independently.

While the scale of our approach has advantages, it may also introduce biases that would be absent in controlled studies. For example, our data are concentrated around areas with a large human presence, such as cities. This introduces a distribution bias towards citizen scientists observing *A. carolinensis* that are in a more urban environment, which has been shown to impact behavior in *Anolis* species [32]. Thermoregulatory behavior has the potential to vary in urban and rural environments, but studying this distinction may be better explored through structured smaller scale observational studies where biases may be better controlled. Additionally, community science sourced images are not standardized in lighting or proximity, which can lead to modest prediction error using the clustering methods employed here. We believe our 93% accuracy was a reasonable trade-off between image retention and correct morph identification.

In sum, the extraordinary growth of citizen field photographs of individual organisms, their crowd-sourced identification, and semi-automated approaches to derive secondary traits from those images is enabling the community to address old questions in news ways [13]. However, these data can still be noisy, requiring careful validation and understanding of biases. We also again reiterate that such macroscale studies are not, by themselves, sufficient to fully answer questions related to causes of biological phenomena such as physiological color change. It is crucial that big data studies work in tandem with traditional experimental and more structured observational studies in order to best uncover context and cues responsible for behaviors such as physiological change [33].

## Data Availability

The dataset, including the segments generated in this study, are available on Zenodo (10.5281/zenodo.15757495). The Python and R scripts used in this study are available on GitHub (https://github.com/sprice03/acarolcolor).

## Abbreviations

AI: Artificial intelligence
RGB: Red green blue
PRISM: Parameter-elevation regressions on independent slopes model
GAM: General additive model
AIC: Akaike information criterion
